# Potentiating Gentamicin Efficacy Against Biofilms of Clinically Relevant Gram-Negative Bacteria Using Biosynthesized ZnO Nanoparticles

**DOI:** 10.3390/pharmaceutics18080913

**Published:** 2026-07-24

**Authors:** Akshit Malhotra, Kwthar Debbarma, Sangita Jana, Irusan Dhinakaran, Surisetty Jaya Prasanthi, Elvira Rozhina, Ram Karan, Ashwini Chauhan

**Affiliations:** 1 Department of Microbiology, University of Delhi South Campus, Benito Juarez Road, New Delhi 110021, India; akshitmalhotra@south.du.ac.in (A.M.); sangita.jana1514@gmail.com (S.J.); ramkaran@south.du.ac.in (R.K.); 2 Department of Microbiology, Tripura University (A Central University), Suryamaninangar, Agartala 799022, India; kwthar567.kd@gmail.com; 3 The Madras Medical Mission, 4-A Dr JJ Nagar Mogappair, Chennai 600053, India; microdhina85@gmail.com (I.D.); gjprasanthi@gmail.com (S.J.P.); 4 Institute of Fundamental Medicine and Biology, Kazan Federal University, Kreml uramı 18, 420008 Kazan, Republic of Tatarstan, Russia; rozhinaelvira@gmail.com

**Keywords:** green synthesis, ZnO nanoparticles, bacterial biofilms, anti-microbial resistance, antibiofilm strategies, nanotoxicology

## Abstract

**Background**: Gram-negative bacteria resistant to multiple drugs are a major cause of illness and death worldwide. Their remarkable capacity to develop resistance to antibiotics makes them a serious concern in medical practice. **Methods**: A simple, green, novel method is used to synthesize ZnO nanoparticles (ZnO NPs) using ethanolic extracts of Diplazium esculentum via precipitation. **Results**: ZnO NPs exhibit a hexagonal structure with a particle size of ~30 nm and a band gap of 3.24 eV. The defect sites formed in ZnO NPs were estimated using prominent peaks in the photoluminescence spectra. ZnO NPs displayed a more than 4-log reduction in multi-drug-resistant *E. coli* and *K. pneumoniae* clinical isolates at a 500 μg/mL concentration. Moreover, ZnO NPs significantly reduced the biofilm bacterial cell viability of clinical isolates of Gram-negative bacteria. Complete eradication of biofilms was achieved for drug-resistant *E. coli* clinical isolates using a combination of sub-MIC of gentamicin and 500 μg/mL ZnO NPs. Green-synthesized ZnO NPs did not induce oxidative stress in mice, as indicated by unchanged GST, GSH, and thiol levels across all the tested organs. ZnO NPs showed both antibacterial and antibiofilm efficacy against drug-resistant strains of *E. coli*, *K. pneumoniae,* and *S. aureus* and completely eradicated *E. coli* biofilm in combination with gentamicin. **Conclusions**: Our study focuses on the sustainable synthesis of biocompatible ZnO NPs for the treatment of infections caused by pathogens belonging to the high-priority ESKAPE group.

## 1. Introduction

The enormous amount of research and development in nanotechnology has led to the development of numerous useful applications, especially in healthcare diagnostics [1], drug delivery [2], and vaccines [3]. The different physical and chemical methods to synthesize nanomaterials involve the use of hazardous and toxic precursors/solvents. Such processes are detrimental to the environment, for example, the use of aromatic chlorinated solvents to synthesize copper [4], silver, and gold nanoparticles [5]. Most of the chemical and physical synthesis approaches to fabricate zinc oxide (ZnO) nanoparticles (NPs) involve environmentally harmful precursor agents, solvents, and reaction byproducts.

Recently, researchers have intensified efforts to develop eco-friendly synthesis processes for advanced nanomaterials [6,7]. Alternative methods based on green chemistry principles [8] are gaining immense impetus among researchers for minimizing the use of hazardous reagents, for instance, the synthesis of AuNPs using an amino acid-containing ionic liquid [9]. Several phytochemical groups have been reported to synthesize a wide array of nanomaterials [10]. Different plant parts, such as roots [11], fruits [12], stems [13], leaves [14], and seeds [15], have been employed to synthesize nanomaterials by exploiting the role of phytochemicals as reducing and stabilizing agents.

Similar to antibiotics, nanomaterials possess the unique ability to target bacterial cell membrane integrity and metabolic machinery [16,17,18]. Metal-based nanoparticles exhibit broad-spectrum antibacterial and antibiofilm activities; therefore, they have been utilized in several biomedical applications [19,20]. Due to the alarming resistance in pathogens against current clinical therapeutics, it is important to develop newer therapeutic solutions. Previously, metal oxide nanoparticles, especially ZnO NPs, have been shown to treat MDR bacteria in combination with antibiotics such as meropenem to eradicate *P. aeruginosa* keratitis in vivo [21]. ZnO NPs potentiated the antibacterial activity of meropenem against carbapenem-resistant *Klebsiella pneumoniae* and down-regulated the expression of genes associated with biofilm formation [22]. However, there is a significant limitation to these studies: reliance on classical minimum inhibitory concentration (MIC) assays to assess antibacterial activity without performing cell viability assays. Assessing cell viability using a colony-forming unit (CFU) assay, i.e., the gold standard for enumerating bacterial cells [23,24], would strongly validate the findings and provide the number of killed and surviving cells.

To address the clinical trend of escalating antibiotic failure due to biofilm-mediated resistance, this study’s objective was to develop a potentiation strategy using ZnO NPs to restore the efficacy of gentamicin. Underpinning environmental sustainability, we describe a novel, green synthesis route using *D. esculentum* extract to produce ZnO NPs. This approach offers significant advantages over chemical synthesis, including enhanced biocompatibility and reduced precursor toxicity, which are critical for clinical translation. We evaluated these NPs against ‘high-priority’ multi-drug-resistant clinical isolates of *K. pneumoniae*, *E. coli*, and *S. aureus*. Our findings demonstrate that ZnO NPs can surpass the limitations of traditional monotherapy; when combined with sub-MIC of gentamicin, they completely eradicate biofilms of clinical *E. coli* isolates. Furthermore, our in vivo assessment addresses the primary disadvantage associated with metallic nanoparticles—potential systemic toxicity—confirming that these biosynthesized NPs are safe for biological applications.

## 2. Materials and Methods

### 2.1. Collection of Plant Parts, Preparation of Extract and Characterization of Plant Metabolites Using LC-HRMS

The plant leaves of *Diplazium esculentum* were washed with DI water and then dried in dark conditions at ambient temperature. After this, the dried leaves were crushed into a powder. For extraction, we added 10 g of powdered sample to 100 mL of ethanol and stirred continuously for 24 h at 150 rpm. Later, the extract was filtered, and the filtrate was concentrated using a rotary evaporator to obtain an ethanolic crude extract. The obtained extract was further lyophilized and stored at 4 °C. The ethanolic extract was characterized using LC-HRMS (Agilent 1290 II–6546 Q-TOF, Agilent, Santa Clara, CA, USA). Metabolites were separated via liquid chromatography, followed by high-resolution mass spectrometry in both positive and negative ionization modes. Data were filtered by mass accuracy (<5 ppm) and aligned with METLIN metabolite database scores.

### 2.2. Synthesis of Diplazium esculentum-Based Zinc Oxide Nanoparticles

Zinc oxide nanoparticles were synthesized via green synthesis approach using *Diplazium esculentum* plant extracts. Briefly, ZnCl_2_, a highly concentrated Zn precursor, was utilized to synthesize ZnO NPs. In this reaction mixture, *Diplazium esculentum* medicinal plant acts as a reducing agent and ZnCl_2_ as a sole source of Zn^2+^ ions. Initially, 95 mL of 0.2 M ZnCl_2_ was prepared, and 5 mL plant extract of *D. esculentum* (concentration 10 mg/mL) was added to it, followed by pH adjustment to 6. After continuous mixing of the total reaction mixture for 1 h, the mixture was centrifuged at 15,000 rpm for 10 min at 4 °C. The supernatant was discarded, and the pellet was resuspended in 50% ethanol (*v*/*v* in distilled water) in a fresh tube. Centrifugation was performed twice, and the pellet was dried in an oven for 12 h at 80 °C to obtain a total yield of 2.25 g.

### 2.3. Characterization of Nanoparticles

Diffraction intensity from *hkl* planes of synthesized NPs was recorded via theta–theta technique employing powder X-ray diffractometer (PANalytical, EMPYREAN, Malvern Panalytical Ltd., Worcestershire, UK). Diffraction pattern was obtained at 45 kV and 40 mA between 20 °C and 80 °C using Cu Kα source (1.5406 Å). Surface morphology and elemental identification were performed using FE-SEM (Sigma-300, Carl Zeiss, Carl Zeiss AG, Oberkochen, Germany) and EDAX, respectively. Diffuse reflectance intensity from the synthesized nanoparticles was recorded over 200 to 800 nm using diffuse reflectance spectroscopy (Lambda-365 UV-Vis Spectrophotometer, Perkin Elmer, PerkinElmer Inc., Norwalk, CT, USA) to determine the band gap. The photoluminescence (PL) emission intensity was recorded at 355 nm and 285 µW incident power employing the Witech Alpha 300 RAS system (WITec GmbH, Ulm, Germany).

### 2.4. Bacterial Strains and Growth Media

The bacterial strains used in this study were clinical isolates of E. coli (ECU-6), *S. aureus* (SAW1), *K. pneumoniae* (KPS), and *K. pneumoniae* (KPP1) obtained from Agartala Government Medical College (AGMC), Tripura. Apart from that, a *K. pneumoniae* MTCC 10309 and fourteen multi-drug resistant (MDR) *K. pneumoniae* strains procured from Madras Medical Mission Hospital, Chennai, Tamil Nadu, were also assessed in this study. All the biological experiments on clinical isolates were conducted in a biosafety level-2 facility after approval of the protocols by the University of Delhi South Campus (IBKP UAC No. UNIRDARB3236), New Delhi, India. All Gram-negative bacteria (ECU-6, KPS, MTCC 10309, KPP1 and *K. pneumoniae* clinical isolates) were grown in Luria–Bertani broth (GM575, HiMedia Laboratories, Kennett Square, PA, USA), and SAW1 was grown in Tryptic Soy Broth (GM011, HiMedia Laboratories) at 37 °C. The bacterial cell count was performed by spotting serially diluted cultures onto sterile TSB agar (SAW1) or LB agar (ECU-6, KPS, MTCC 10309, KPP1, and *K. pneumoniae* clinical isolates) plates and incubating for 12–16 h at 37 °C.

### 2.5. Evaluation of In Vitro Bacterial Cell Viability by Exposing Exponential Phase Bacteria to Diplazium esculentum-Based ZnO NPs

After growing overnight, the bacterial strains were sub-cultured to obtain exponential phase bacterial cells, which were then treated with ZnO NPs at varying concentrations (250, 500, 750 μg/mL). The treated bacterial cultures were allowed to shake continuously at 850 rpm for 24 h at 37 °C in Eppendorf ThermoMixer^®^ C (Eppendorf SE, Hamburg, Germany). The untreated bacterial cultures were used as controls. After 24 h, the treated and untreated bacterial cultures were serially diluted, spotted onto Luria agar (ECU6 and KPP1) or TSB agar (SAW1) plates, and incubated for 12–16 h at 37 °C. All the experiments were performed at least in triplicate. The antibacterial activity of ZnO NPs was evaluated against fourteen MDR *K. pneumoniae* clinical isolates (KPS1, ON24, ON4400, ON1080, ON9569, ON5215, ON4380, O760, O2697, O3875, O3283, O2040, B864, B936, and B776) and *K. pneumoniae* MTCC 10309. All bacterial strains were routinely cultured in Luria–Bertani (LB) broth at 37 °C with shaking at 150 rpm.

### 2.6. In Vitro Evaluation of AntiBiofilm Activity by Diplazium esculentum-Based ZnO NPs

The biofilms were grown in 96-well microtiter plates as described earlier [25]. Briefly, the overnight-grown bacteria were inoculated into sterile LB Broth (ECU6 and KPP1) or TSB Broth (SAW1) at a 1:100 dilution in microtiter plate wells for 24 h at 37 °C to form biofilms under static conditions. Before treating the biofilms, the planktonic, non-adherent bacteria were removed and discarded from each well, followed by three washes with 1× PBS. The biofilms adhering to the well surfaces were treated with different concentrations of ZnO NPs (250, 500, and 750 μg/mL). The untreated control wells received 1×PBS. After 24 h treatment, the biofilm bacterial cell viability was determined by serially diluting the well contents and plating the dilutions on LB agar plates (ECU6 and KPP1) or TSB agar plates (SAW1). The colonies were counted to calculate viable CFU/mL in each treatment and compared with untreated controls.

### 2.7. In Vitro Potentiation of Gentamicin to Eradicate Gram-Negative and Gram-Positive Bacterial Biofilms

We determined minimum inhibitory concentration (MIC) values of gentamicin for different bacteria, used in this study, via broth microdilution assay, as done previously [26]. In order to assess the potentiation ability of ZnO NPs, ZnO nanoparticles (250 or 500 μg/mL) were taken in combination with sub-MIC concentration of gentamicin to treat the 24 h old bacterial biofilms, and the biofilm bacteria cell viability was checked after 24 h of treatment. ZnO NPs alone or gentamicin alone was used to compare the results. The viable colonies were observed to calculate CFU/mL in each treatment.

### 2.8. Mode of Action of Diplazium esculentum-Based ZnO NPs

The intracellular reactive oxygen species (ROS) levels in ECU6 and KPP1 strains were quantified following treatment with ZnO nanoparticles (ZnO NPs), gentamicin, and their combination, using 2′,7′-dichlorodihydrofluorescein diacetate (DCFH-DA). Exponentially growing bacterial cultures were exposed to ZnONPs at a concentration of 500 μg/mL, gentamicin at a sub-MIC concentration, and incubated under shaking conditions at 37 °C for 24 h. Post treatment, the bacterial cells were pelleted via centrifugation at 10,000 rpm for 10 min and washed once with PBS to remove residual media. The cell pellets were resuspended in PBS containing 1 mM DCFH-DA and incubated in the dark at 37 °C for 30 min. DCFH-DA is a non-fluorescent compound that, upon intracellular deacetylation and oxidation by ROS, converts into the fluorescent compound 2′,7′-dichlorofluorescein (DCF). Fluorescence intensity, indicative of ROS generation, was measured using a BioTek Synergy H1 microplate reader (BioTek Instruments, Inc., Winooski, VT, USA) at an excitation wavelength of 488 nm and an emission wavelength of 535 nm [27].

### 2.9. In Vivo Toxicology Assessment of Diplazium esculentum-Based ZnO NPs

Male Balb/C mice aged 6–8 weeks were procured from Zydus Research Center, Ahmedabad. All mice were housed in the animal care center under a 12 h light/dark cycle, with environmental conditions maintained at 22 °C ± 1 °C and 55% ± 10% humidity. Water and autoclaved food (standard rodent diet) were given ad libitum. The mice were acclimatized for 1 week prior to the experiments. After one week, the mice were segregated into different groups; each group consisted of three mice. All mice in different treatment groups received a single dose of 50 μg of nanoparticles in 100 μL 1× PBS. The control group received 100 μL 1× PBS instead of nanoparticles. Every day, each mouse was weighed and monitored for any changes. To examine the cellular effect or pathological events, all mice were euthanized after blood withdrawal after 7 days of treatment. Organs, including the brain, lungs, liver, spleen, testes and kidneys, were harvested. The organs were fixed in 10% formalin and subjected to further histopathological examinations.

### 2.10. Statistical Analysis

All experiments were performed in triplicate (*n* = 3), and results are expressed as the mean ± standard deviation (SD). Statistical significance was determined using GraphPad Prism (version 8.0.1). To compare multiple treatment groups and concentrations for antibacterial, antibiofilm, and intracellular ROS generation assays, Welch’s ANOVA followed by the Games–Howell multiple comparisons test was performed. This statistical framework was selected following a Brown–Forsythe test to account for the non-homogeneity of variance across experimental groups. Probability values of *p* < 0.05 were considered statistically significant (* *p* < 0.05, ** *p* < 0.005, *** *p* < 0.0005, **** *p* < 0.00005).

## 3. Results

### 3.1. Phytochemical Profiling in Diplazium esculentum Plant Extract

The phytochemical profile of the *Diplazium esculentum* ethanolic extract was characterized by a high abundance of the triterpenoid Cucurbitacin E, suggesting significant anti-inflammatory potential. The identification of key antioxidant markers, including Eudesmin, Eriodictyol, and Kaempferol glycosides, corroborates the plant’s high radical scavenging capacity. Furthermore, the preservation of volatile terpenes such as Sabinene and Phytol underscores the efficacy of the extraction process in maintaining a broad spectrum of bioactive secondary metabolites.

### 3.2. Diplazium esculentum-Mediated ZnO NPs Exhibited Hexagonal Phase with Wurtzite Structure

**Figure 1:**
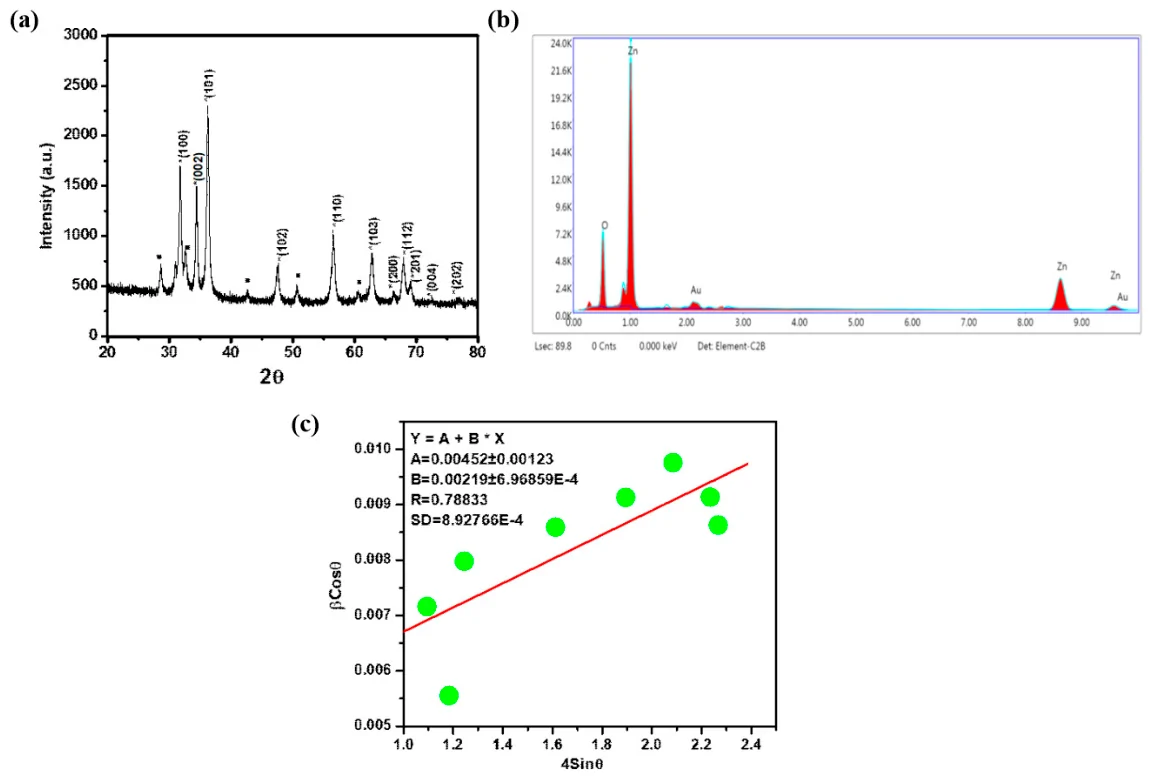
(**a**) X-Ray diffraction pattern from the *Diplazium esculentum*-based phyto-assisted precipitation-synthesized NPs. Here, star symbol represents ZnO phase and solid square represents intermediate phase. (**b**) EDAX measurement for the synthesized NPs, indicating only *Zn* ad *O* signals (Au peak corresponds to the gold deposition done over the sample for FE-SEM scanning). (**c**) Williamson–Hall plot for the *Diplazium esculentum*-based phyto-assisted precipitation-synthesized ZnO NPs.

ZnO NPs synthesized using *Diplazium esculentum* showed diffraction peaks from various planes, as identified with the help of JCPDS file 36–1451 (Figure 1a). The nanoparticles exhibited a hexagonal phase with a wurtzite structure. There were some low-intensity peaks in addition to those characteristic of ZnO. EDAX spectroscopy (Figure 1b) confirmed the elemental composition comprising Zn and O elements, therefore ruling out the formation of other byproducts. Thus, the low-intensity peaks in the XRD pattern could be attributed to intermediate phases formed during ZnO nanomaterial synthesis, as reported previously by several researchers [7,28,29]. The average crystallite size of the ZnO NPs was determined to be 30.67 nm using a Williamson–Hall (Figure 1c) plot, as described previously [30,31]. An indexing program [32] was adopted to determine lattice parameters ‘a’ and ‘c’ as 3.252 Å and 5.214 Å, respectively, at R-factor= 0.00001 and F~25.6. The high F-value (25.6) and exceptionally low R-factor (0.00001) confirm the high precision of the regression model used for calculating lattice parameters, consistent with standard crystallographic validation.

### 3.3. Synthesized ZnO NPs Showed Spherical Surface Morphology

**Figure 2:**
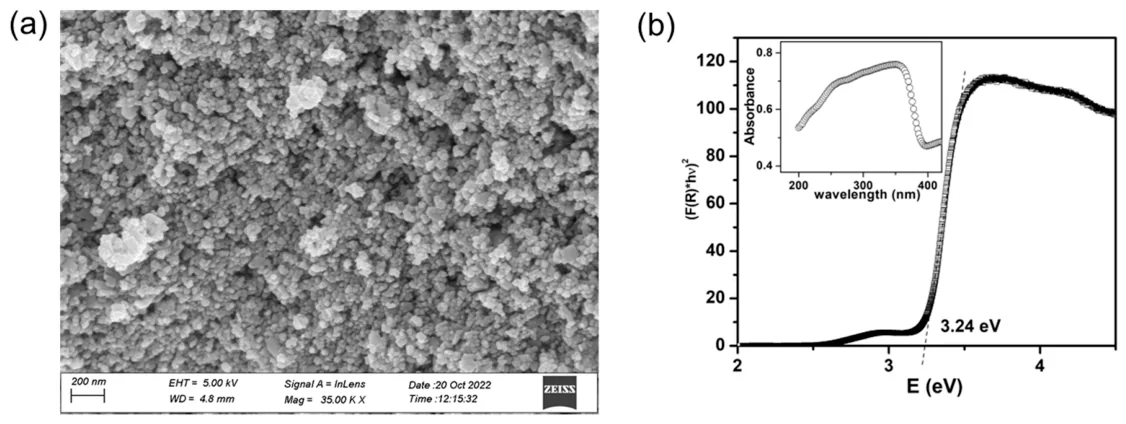
(**a**) FE-SEM image of nanoparticles reveals formation of homogenous particles. (**b**) ${\left[F\left(R\right)h\nu \right]}^{2}$ versus hν plot for determining band gap. Intercept yield for the band gap of *Diplazium esculentum*-based phyto-assisted precipitation-synthesized NPs; inset shows the absorption spectra for the synthesized NPs.

FE-SEM revealed spherical morphology (Figure 2a) of ZnO NPs synthesized using *Diplazium esculentum* ethanolic extract. The mean particle size of the synthesized NPs as obtained via FE-SEM was ~31.9 nm. Further, UV-Vis and diffuse reflectance spectroscopy showed that the band gap E_g_ of ZnO NPs synthesized using *D. esculentum* was measured as 3.24 eV (Figure 2b) using the Kubelka–Munk (KM) relation, as described earlier [33,34]. The absorption peak for ZnO NPs synthesized using *D. esculentum* was observed at 376 nm in the absorption spectra displayed by ZnO NPs (Figure 2b), inset).

### 3.4. Photoluminescence (PL) Spectroscopy Analysis

**Figure 3:**
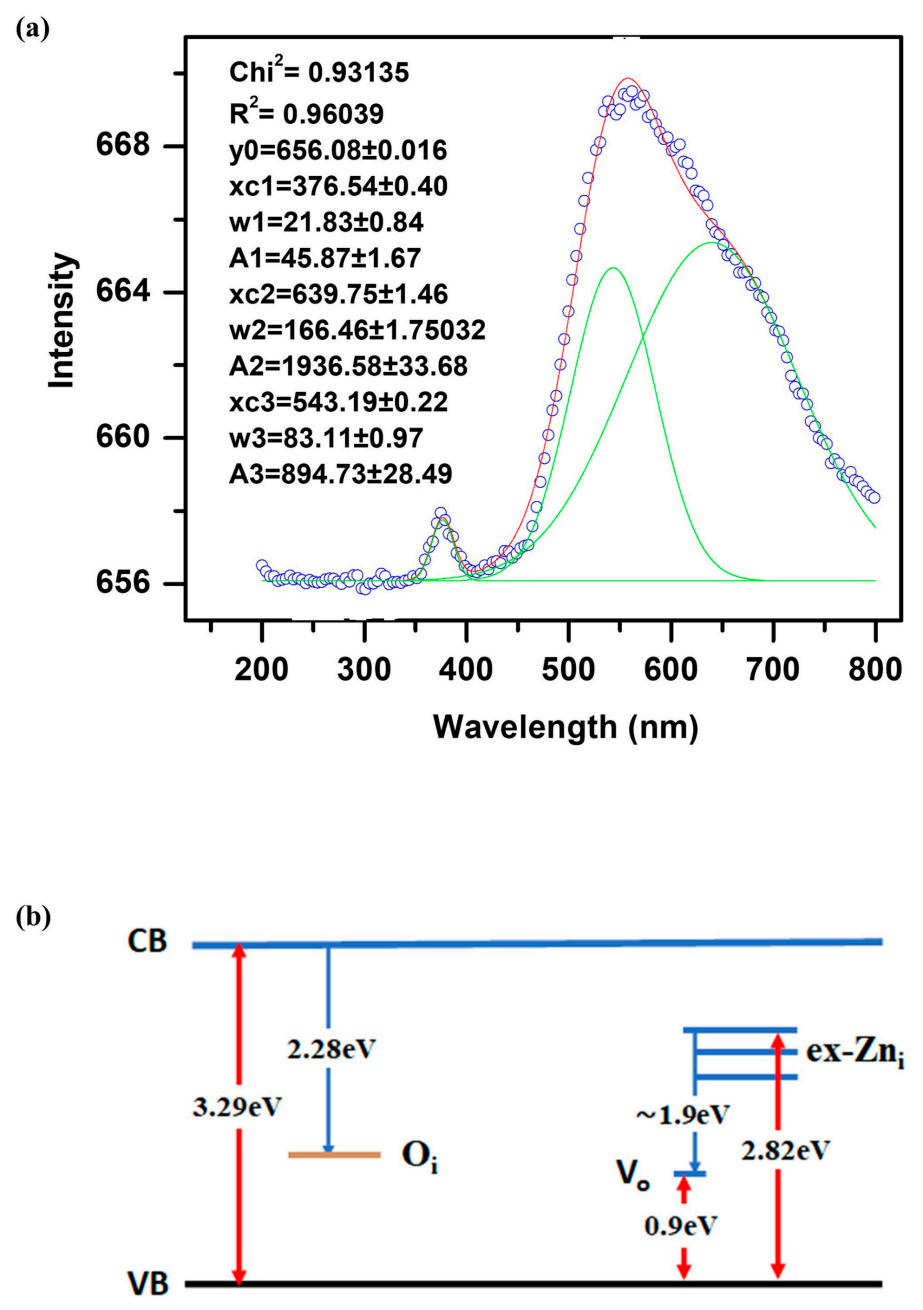
(**a**) RT-PL from *Diplazium esculentum*-based phyto-assisted precipitation-synthesized ZnO NPs. (**b**) Possible energy level diagram for synthesized ZnO NPs using *Diplazium esculentum*. The green emission could be due to electronic transition occurring from CB to O_i_ level. The orange–red emission may be due to ex-Zn_i_ and oxygen vacancy levels.

The defect states present in the synthesized sample were investigated via room-temperature photoluminescence (RT-PL) spectroscopy (Figure 3a). The emission band at 376 nm corresponds to near-band-edge UV emission arising from band-to-band recombination. In addition to this feature, a broad and intense visible emission band was observed, indicating the presence of multiple defect states within the ZnO lattice.

To elucidate the nature of these defects, the broad visible emission band was deconvoluted using Gaussian fitting (Figure 3a). The deconvolution revealed multiple emission components underlying the broad peak. Specifically, the PL spectrum exhibited a strong orange–red emission centered at 639.75 nm (1.94 eV) [35] and green emission at 543.19 nm (2.28 eV) [36,37,38,39], as shown in the band diagram (Figure 3b). As the Gaussian deconvolution was performed with a high correlation coefficient (R^2^ > 0.99), the identified peak centers correctly represent the dominant defect states in the ZnO lattice.

The green emission is commonly attributed to oxygen interstitial (O_i_) defects [36], corresponding to electronic transitions from CB to O_i_ defect levels [35]. In contrast, the orange–red emission is likely associated with defect states arising from ex-Zn_i_ [40] and oxygen vacancies [37,40]. Collectively, these defect-related emissions indicate the presence of intrinsic point defects that contribute to the visible photoluminescence of the synthesized ZnO nanoparticles.

### 3.5. Zinc Oxide Nanoparticles Showed Antibacterial Activity Against Clinical Isolates of Gram-Negative and Gram-Positive Bacteria

**Figure 4:**
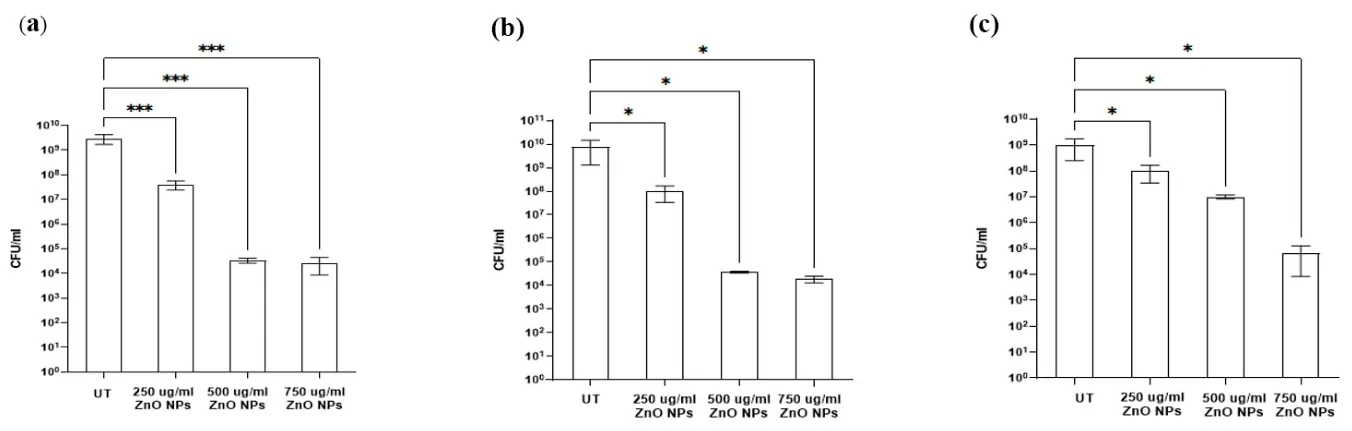
Antibacterial activity of *Diplazium esculentum*-based ZnO NPs against (**a**) *E. coli* ECU6, (**b**) *K. pneumoniae* KPP1, (**c**) *S. aureus* SAW1 with different concentrations 250–750 μg/mL after 24 h exposure. Statistical significance was determined using Welch’s ANOVA followed by the Games–Howell multiple comparisons test (GraphPad Prism, version 8.0.1). Differences were considered significant at *p* < 0.05 *, *p* < 0.0005 ***. UT—Untreated, GENT—Gentamicin, ZnO NPs—Zinc Oxide Nanoparticles.

In the present study, the inhibitory nature of ZnO NPs was evaluated by treating actively growing bacteria with different concentrations (250 μg/mL to 750 μg/mL) of ZnO NPs, wherein bacteria treated with 1× PBS were used as controls (Figure 4). At 250 μg/mL concentration of NPs, we found close to a 2-log reduction for *E. coli* ECU6 (Figure 4a) and *K. pneumoniae* KPP1 (Figure 4b) but only a modest 1-log reduction in the growth of SAW1 (Figure 4c). At 500 μg/mL concentration of NPs, we found a 5-log reduction in the growth of ECU6 and KPP1 but a 2-log reduction in the growth of SAW1. Although further increasing the ZnO NP concentration to 750 μg/mL led to no change in the killing efficiency against Gram-negative bacteria, a 4-log reduction in the growth of *S. aureus* SAW1 was observed.

The antibacterial efficacy of ZnO nanoparticles against *K. pneumoniae* MTCC 10309 strain and fifteen clinical isolates of *K. pneumoniae* was evaluated by determining the percentage reduction in viable cell count following 24 h exposure to increasing nanoparticle concentrations (50–750 µg/mL). As shown in Supplementary Figure S1, across the majority of isolates, ZnO nanoparticles induced a clear concentration-dependent antibacterial effect. At lower concentrations (50 and 100 µg/mL), variable susceptibility was observed among isolates, with percentage killing ranging from minimal to moderate levels. Certain isolates (ON24, O3875, and B936) displayed low sensitivity at these concentrations, whereas others (ON4400, O760, and ON9569) exhibited an approximately 50% reduction in viable cells. At an intermediate concentration (250 µg/mL), an increase in antibacterial activity was observed for most isolates, with several strains showing >80–90% killing. ZnO NP treatment at higher concentrations (500 and 750 µg/mL) resulted in near-complete or complete bacterial killing (~95–100%) in the majority of clinical isolates and the reference MTCC 10309 strain. Overall, the results demonstrate that ZnO nanoparticles possess strong bactericidal activity against *K. pneumoniae*, with efficacy increasing in a dose-dependent manner and achieving complete eradication in few isolates.

### 3.6. ZnO NPs Inhibited Biofilms Formed by Clinical Isolates of Bacteria

**Figure 5:**
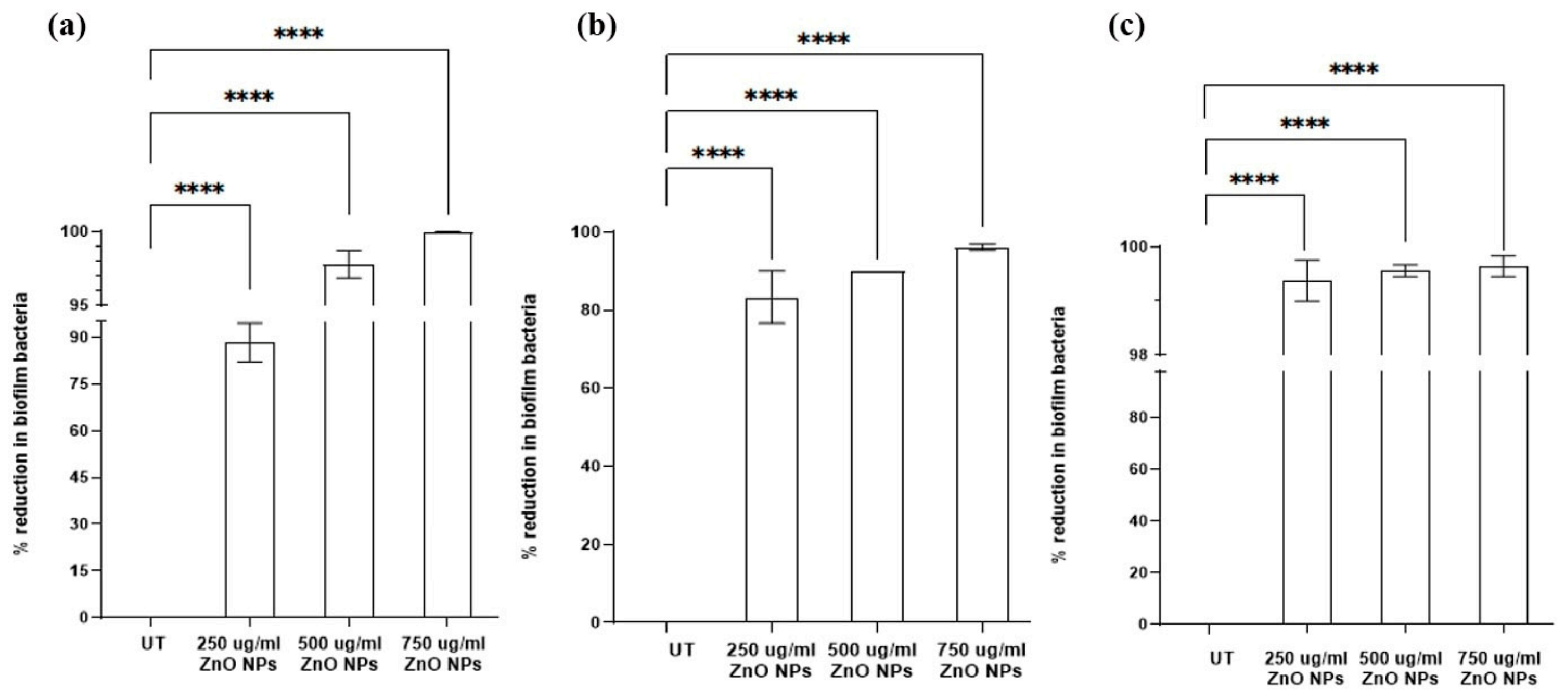
Antibiofilm activity of *Diplazium esculentum*-based ZnO NPs against (**a**) *E. coli* ECU6, (**b**) *K. pneumoniae* KPP1, (**c**) *S. aureus* SAW1 with different concentrations 250–750 μg/mL after 24 h exposure of NPs. Statistical significance was determined using Welch’s ANOVA followed by the Games–Howell multiple comparisons test (GraphPad Prism, version 8.0.1). Differences were considered significant at *p* < 0.0001 ****. UT—Untreated, ZnO NPs—Zinc Oxide Nanoparticles.

In our study, biofilms of ECU6, KPP1 and SAW1 were grown in a 96-well microtiter dish using Luria–Bertani broth or Tryptic Soy Broth for 24 h. Further, different treatments of ZnO NPs (250–750 μg/mL) were given along with fresh media, followed by incubation for 24 h. We found a significant reduction in viable cells in the biofilm for all bacterial strains (Figure 5). ZnO NPs were able to eradicate 97% *E. coli* ECU6 (Figure 5a), 90% *K. pneumoniae* KPP1 (Figure 5b), and 99.5% *S. aureus* SAW1 biofilm bacteria cells at a concentration of 500 μg/mL (Figure 5c). We found 500 μg/mL to be the optimum concentration as it did not show in vivo oxidative stress and was effective at eradicating biofilms and was, thus, selected to potentiate the antibiofilm activity of aminoglycoside.

### 3.7. ZnO NPs Potentiated the In Vitro Biofilm Eradication via Aminoglycoside Against Gram-Negative Biofilms

**Figure 6:**
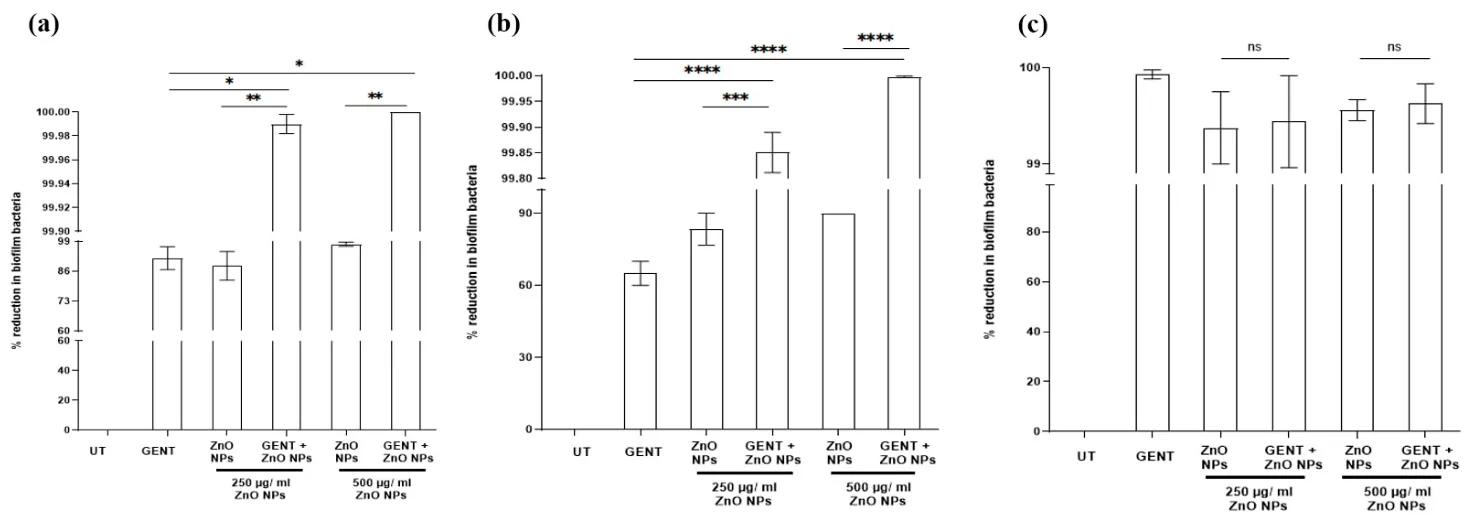
Combination study of *Diplazium esculentum*-based ZnO NPs with gentamicin against (**a**) *E. coli* ECU6, (**b**) *K. pneumoniae* KPP1, (**c**) *S. aureus* SAW1 with different combinations after 24 h exposure. Statistical significance was determined using Welch’s ANOVA followed by the Games–Howell multiple comparisons test (GraphPad Prism, version 8.0.1). Differences were considered significant at *p* < 0.05 *, *p* < 0.005 **, *p* < 0.0005 ***, *p* < 0.0001 ****. UT—Untreated, GENT—Gentamicin, ZnO NPs—Zinc Oxide Nanoparticles, ns—not significant.

We determined the MIC of gentamicin for all bacterial isolates using the standard broth microdilution method, as done earlier [26]. We found the MIC of gentamicin against *E. coli* ECU6, *S. aureus* SAW1, and *K. pneumoniae* KPP1 to be 3.125 μg/mL, 6.25 μg/mL and 1.56 μg/mL, respectively. We treated 24 h old biofilms with a sub-MIC concentration of gentamicin in combination with 250 or 500 μg/mL of NPs. The gentamicin-ZnO NP combination showed synergistic antibiofilm activity against Gram-negative bacteria, whereas an antagonistic effect was observed against Gram-positive bacteria. The sub-MIC of gentamicin (1.56 μg/mL) reduced the viable bacterial cell population in the *E. coli* ECU6 biofilm only by 91%, whereas, comparatively, when gentamicin (1.56 μg/mL) was combined with ZnO NPs (250 μg/mL), it reduced the viable bacterial count of the *E. coli* ECU6 biofilm by 99.99%. Remarkably, complete eradication of the *E. coli* ECU6 biofilm was achieved when the concentration of ZnO NPs was increased to 500 μg/mL in combination with sub-MIC of gentamicin (Figure 6a). In the case of *K. pneumoniae* KPP1 biofilms, using ZnO NPs (250 μg/mL) along with a sub-MIC of gentamicin (0.78 μg/mL) resulted in a 99.8% reduction in biofilm bacterial cell viability. Increasing the ZnO NP concentration 2× (500 μg/mL) in combination with sub-MIC of gentamicin improved the antibiofilm efficacy to 99.996% (Figure 6b). However, we observed an antagonistic effect when gentamicin and ZnO NPs were used in combination to treat *S. aureus* SAW1 biofilms (Figure 6c). The antibiofilm activity at a sub-MIC concentration of gentamicin was better than the combination of gentamicin at MIC and ZnO NPs for *S. aureus* SAW1. Further, detailed further study is required to elucidate the mechanism for this antagonism, unlike Gram-negative bacteria.

### 3.8. Intracellular Reactive Oxygen Species Production in Response to ZnO NP and Gentamicin Treatment

**Figure 7:**
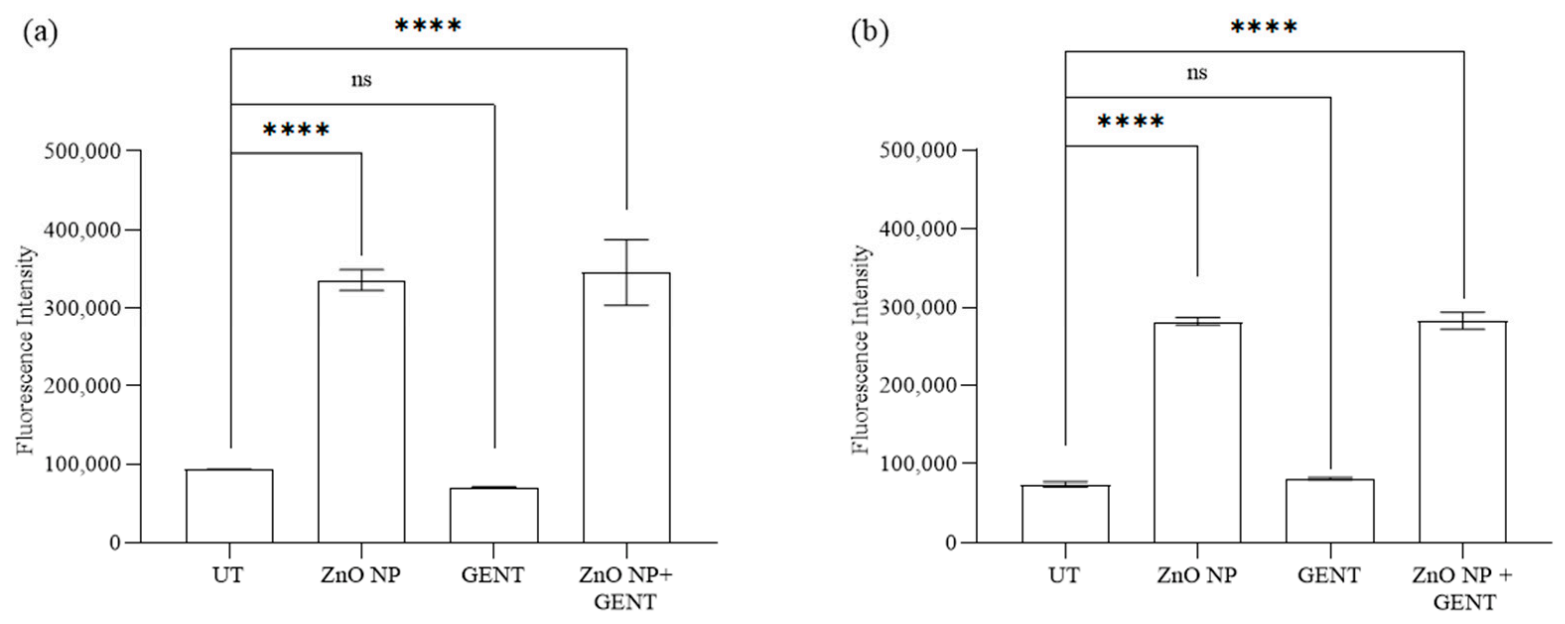
Estimation of reactive oxygen species (ROS) generated by (**a**) *Escherichia coli* ECU6 and (**b**) *Klebsiella pneumoniae* KPP1 in response to the treatment of ZnO NPs (500 µg/mL), gentamicin (1.5 µg/mL for ECU6 and 0.78 µg/mL for KPP1), and their combination after 24 h of exposure. Statistical significance was determined using Welch’s ANOVA followed by the Games–Howell multiple comparisons test (GraphPad Prism, version 8.0.1)). Differences were considered statistically significant at *p*-value < 0.05 (**** represents *p*-value < 0.0001, UT—untreated control; GENT—gentamicin; ZnO-NPs—zinc oxide nanoparticles; ns—not significant).

The intracellular reactive oxygen species (ROS) generation in *E. coli* ECU6 and *K. pneumoniae* KPP1 upon treatment with green-synthesized ZnO NPs, gentamicin and their combination was quantified using the DCFH-DA assay. In both strains, a significant increase in ROS generation was observed upon treatment with ZnO NPs compared to untreated bacteria. In contrast, gentamicin-treated bacteria did not elevate ROS levels. The combination of ZnO NPs and gentamicin produced ROS levels comparable to those of ZnO NPs alone, indicating no synergistic effect on ROS production (Figure 7). These findings demonstrate that ZnO NPs independently induce intracellular ROS production in test bacteria, hence confirming the oxidative mechanism of antibacterial activity. Gentamicin, an aminoglycoside, did not significantly contribute to ROS generation, consistent with its primary mode of action: disruption of protein synthesis by binding to the 30S ribosomal subunit. Notably, the combination of ZnO NPs and gentamicin did not further elevate ROS levels compared to ZnO NPs alone, indicating that gentamicin does not amplify or suppress the oxidative stress induced by ZnO NPs.

### 3.9. Zinc Oxide Nanoparticles Did Not Increase Oxidative Stress and Were Biocompatible In Vivo

**Figure 8:**
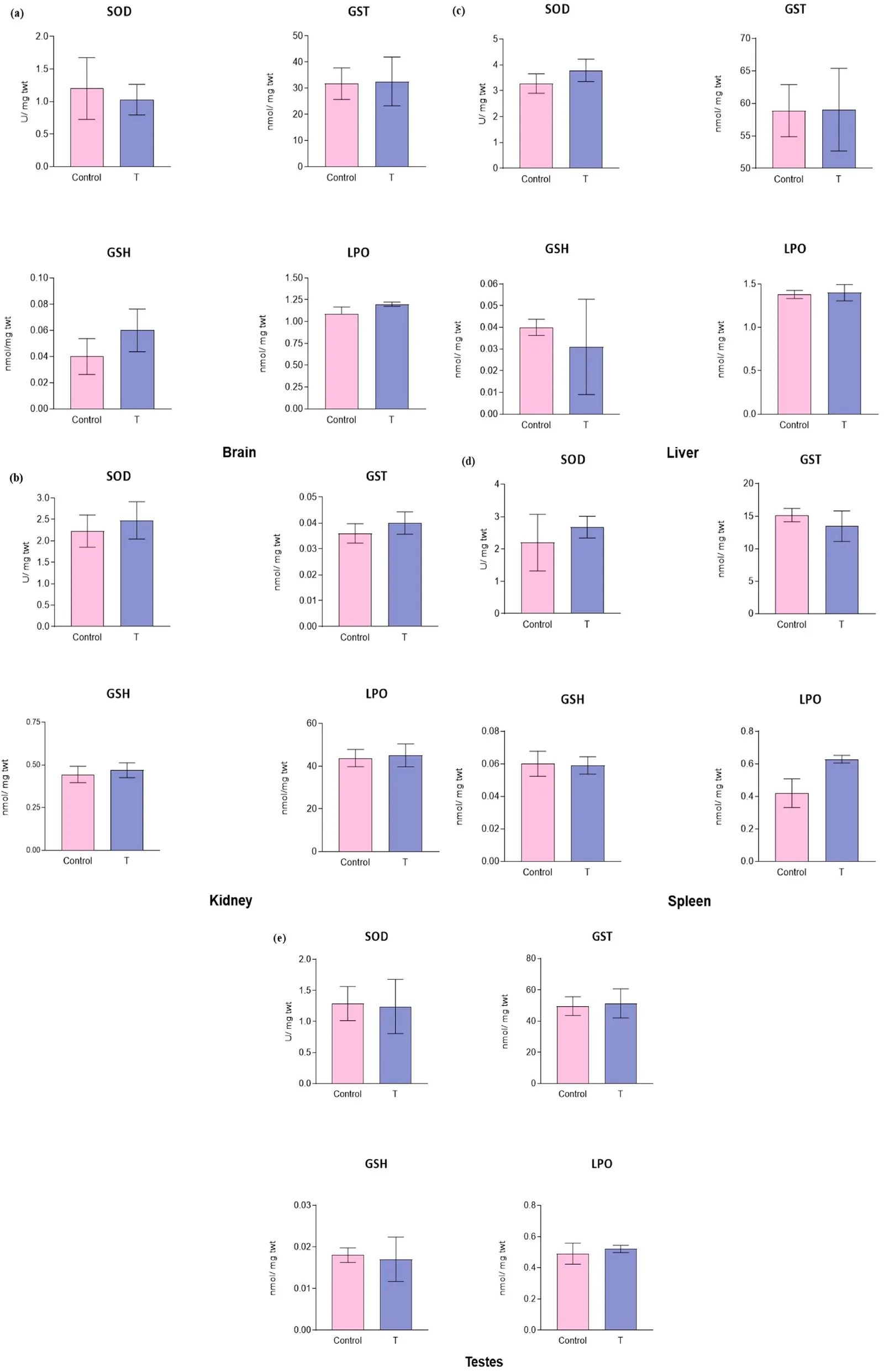
Estimation of oxidative stress parameters in different tissues of mice injected in vivo. (**a**) Brain. (**b**) Kidneys. (**c**) Liver. (**d**) Spleen. (**e**) Testes.

Oxidative stress parameters were measured in the different tissues, viz. liver, kidneys, lungs, testes and brain, harvested from the mice injected with (treatment group) or without (vehicle control) nanoparticles. With regard to the lipid peroxidation (LPO) in the tissues of animals, it was observed that the levels of MDA were similar in all tissues. Additionally, we examined GST activity, GSH levels, and total thiol levels (Figure 8). Up to 7 days of treatment, there was no passive behavior noticed in mice such as arching of the back, tremor and hypopnea or any poisoning-associated symptoms such as diarrhea, loss of appetite and vomiting. There was no significant difference in the change in body weight during the experimental period among those mice. No significant change in GST activity or GSH levels was observed in any of the tissues examined in the treatment group compared with the vehicle control group. Similarly, the total thiol level remained unchanged in all tissues assessed compared with the vehicle control. No change in any of the oxidative parameters indicated that at the tested concentration of the green-synthesized ZnO NPs, *Diplazium esculentum* did not induce any oxidative stress in the mice and, hence, is safe for use in clinical settings.

## 4. Discussion

Escalating antibacterial resistance poses a critical public health threat. Addressing this requires innovative infection treatment strategies, particularly targeting biofilms, which are notoriously resistant to conventional antibiotics. Zinc oxide nanoparticles (ZnO NPs) offer a promising avenue due to their biocompatibility, cost-effectiveness, and broad antibacterial activity. However, traditional chemical synthesis of ZnO NPs poses environmental risks. Our research demonstrates that eco-friendly, green-synthesized ZnO NPs effectively inhibit biofilm formation in drug-resistant clinical strains, both Gram-positive and Gram-negative. Furthermore, we show the enhanced efficacy of ZnO NPs in combination with gentamycin for bacterial infection control. The ethanolic extract of *Diplazium esculentum* leaves was used to synthesize ZnO NPs through precipitation. The strong correlation between particle sizes derived from XRD and FE-SEM indicates high sample homogeneity, consistent with previous reports of green-synthesized ZnO NPs [40,41] exhibiting uniform particle size distribution. The characterization of the phytochemical profile of *Diplazium esculentum* revealed the dominance of triterpenoids (Cucurbitacin E) and lignans (Eudesmin), alongside a diverse array of phenolics (6-Gingerol), flavonoids (Eriodictyol glycosides), terpenes (Sabinene), and sterol glycosides (Table S2). These bioactive classes may serve as the primary reducing and stabilizing agents essential for the biogenic synthesis of ZnO nanoparticles. The synthesis approach is “atom-economical” and “green” as it replaces the use of toxic chemical stabilizers (CTAB or SDS) and use of large quantities of harsh bases (like NaOH) with natural plant extracts. Specifically, phytochemicals such as triterpenoids, flavonoids, and phenolics facilitate the green reduction and capping of ZnO precursors into stable, bioactive nanoparticles [42]. The high oxygen density in Cucurbitacin E provides multiple coordination sites. The hydroxyl (-OH) and carbonyl (=O) groups can facilitate the reduction of zinc chloride precursors and stabilize the ZnO crystal lattice during nucleation [43]. The preferential adsorption of these functional groups onto the nanoparticle surface not only controls crystal growth but also provides the steric and electrostatic stabilization required to maintain the structural integrity and biocompatibility of the final synthesized nanomaterial.

The ZnO nanoparticles synthesized in this study exhibited sizes comparable to those reported by Prasad et al. (30–45 nm) using *Withania somnifera* root extract [44]. Nevertheless, they were larger than ZnO nanoparticles synthesized using orange peel extract (10–20 nm) [45] and smaller than other biologically synthesized ZnO nanoparticles reported in the literature (45–150 nm) [46]. Furthermore, the observed reduction in the band gap relative to the bulk ZnO (~3.3 eV) is likely attributable to structural defects, such as Zn and O vacancies [47], Zn and oxygen interstitial [48], dislocations [49] and stacking faults [50].

In PL studies, previous reports have shown both red [51,52] and blue shifts [53,54] for ZnO NPs. Marotti et al. attributed the red shift to the crystallite size of ZnO grown via potentiostatic electrodeposition [52], whereas Tan et al. attributed the blue shift to the presence of different phases, specifically the amorphous phase in ZnO thin films [53].

PL peaks originate as a result of recombination from surface states [55]. Particle size [56], surface morphology [57,58], and synthesis process [37] may alter the band gap and introduce defects in the material. In RT-PL, a generally sharp transition is seen in the UV range due to band-to-band transition, whereas the broad emission is pertaining to defects [7]. The presence of more than one fitted peak under the observed broad peak indicated the presence of multiple defect states in the synthesized sample. The presence of multiple peaks in the visible region is a typical feature of nano-crystalline ZnO [7,37].

ZnO NPs exert their antibacterial effect through various mechanisms such as release of Zn^2+^ ions, bacterial cell membrane rupture upon contact and killing of bacterial cells using free radical species [59]. ZnO NPs show broad-spectrum antibacterial and antibiofilm efficacies [60]. In addition, various kinds of doping such as Fe doping [61], different synthesis conditions such as biogenic or chemical route of synthesis [62] and morphological features like hierarchical structures [63] and a high aspect ratio nanorods [64] of ZnO NPs influence their bacterial killing ability and display differential activities for Gram-negative or Gram-positive bacteria. The observed antimicrobial efficacy shows a clear dependence on the nanoparticle’s physicochemical profile. Specifically, the high density of intrinsic lattice defects (oxygen vacancies and interstitials) identified via PL spectroscopy (Figure 3a) likely serves as active sites for the catalytic generation of intracellular ROS (Figure 7) [59]. Previously, several environmentally friendly green approaches were employed to fabricate ZnO NPs and evaluate their biocidal efficacy [48]. However, most studies employ the Kirby–Bauer disk diffusion method, agar well diffusion and determination of minimum inhibitory concentrations using the broth microdilution method. These methods may not be accurate as NPs settle down in static conditions, therefore necessitating continuous shaking to ensure homogenous dispersion of NPs for active surface contact with bacterial cells. Some of the earlier studies determined antibacterial efficacy under shaking conditions [64,65]. In our results, this differential antibacterial activity may be due to the presence of secondary bioactive metabolites (such as flavonoids, phenolics, alkaloids and steroids) in *D. esculentum* that may be more active against Gram-negative bacteria [66]. Although limited, the literature suggests broad-spectrum activity for the *D. esculentum* plant extracts [67,68]. However, detailed investigations and focused studies are needed to ascertain the possible mechanism for the differential activity of *Diplazium esculentum*-based ZnO NPs, which may be due to the role of differential antibacterial activity of phytochemicals present in *Diplazium esculentum*. We observed that 500 μg/mL ZnO NPs, synthesized using *Diplazium esculentum*, were an optimum dosage for *E. coli* ECU6 and *K. pneumoniae* KPPI, whereas, for *S. aureus,* the dosage may be further increased to achieve enhanced activity.

Bacteria utilize their key virulence mechanisms, i.e., the ability to form biofilms [69] and multi-drug resistance, altogether rendering last-resort antibiotics ineffective [70] for a range of infections. The discovery of newer non-antibiotic solutions or alternatives to antibiotics is gaining momentum [71]. Nanoparticles are emerging as promising antibacterial and antibiofilm agents [72]. Previous reports have shown antibacterial and anti-quorum sensing activity of ZnO NPs against multi-drug-resistant *P. aeruginosa* [73].

In our study, we established a significant reduction in biofilm-viable cells in *E. coli, K. pneumoniae* and *S. aureus.* Moreover, ZnO NPs, in combination with sub-MIC concentration of gentamicin, killed 100% of the *E. coli* biofilm and significantly reduced the *K. pneumoniae* biofilm. This enhanced efficacy is attributed to the ability of ZnO NPs to act as membrane-permeabilizing agents. By releasing Zn ^2+^ ions [59] and generating reactive oxygen species (ROS) [60], the efficacy of the antibiotic was improved. However, the combination therapy showed an antagonistic effect on *S. aureus.* Previously, gentamicin was coated on ZnO NPs to treat MDR *E. coli* [74], which resulted in an increase in filamentation and cell damage. However, they did not assess the activity against the biofilm. A 2-fold increase in biofilm inhibition has previously been reported, where ZnO NPs were used in combination with a range of antibiotics for treating *S. aureus* ATCC 6538 biofilms [75]. Moreover, the combination was effective in promoting wound healing in vivo. However, a quantitative reduction in infection was not demonstrated.

The synthesized nanoparticles showed good biocompatibility in an in vivo mouse model, further indicating their safety for biomedical applications. ZnO NPs induced a significant increase in SOD levels in the mouse brain (Figure 7), indicating the absence of reactive oxygen species that inhibit SOD levels, as shown earlier [76]. In other tissues, the levels of the investigated biomarkers of oxidative stress were within the normal range and were concordant with those of the control. It should be noted that the exact mechanisms of the toxic effects of nanoparticles are complex, and, according to the literature, zinc oxide nanoparticles have antioxidant activity with respect to the mechanisms of both free radical scavenging and reducing activity [77,78]. Furthermore, Cucurbitacin E, a prominent bioactive constituent of *Diplazium esculentum*, has been successfully used as a stabilizing and capping agent in the synthesis of polydopamine nanoparticles [79] and silica nanoparticles [80], where it has demonstrated significant anti-inflammatory properties and negligible cytotoxicity. Given this established bioactivity, it is highly probable that *Diplazium esculentum*-mediated nanoparticles do not induce in vivo stress in the mouse model. Consequently, our findings suggest that these ZnO NPs, at the tested concentrations, are safe for potential clinical applications, although the consequences of chronic, long-term exposure warrant further investigation.

## 5. Conclusions

In the pipeline of non-antibiotic-based therapeutics to combat medical biofilms, nanoparticles are increasingly explored for their multi-dimensional arsenal to invade biofilms. The current study underpins the sustainable approach to synthesize biocompatible ZnO NPs and treat drug-resistant bacterial infections. This is the first pursuit to synthesize biocompatible ZnO NPs using *Diplazium esculentum* medicinal plant extracts and efficiently kill clinical isolates of drug-resistant bacteria. Moreover, complete eradication of the clinical isolate of *E. coli* was achieved, in addition to the more than 4-log killing of the *K. pneumoniae* clinical isolate by potentiating the activity of aminoglycoside. There are very few reports outlining the potentiation of existing antibiotics using ZnO NPs. Indeed, restoring the susceptibility of drug-resistant bacteria using ZnO NPs needs intensive research attention. It is equally important to understand the mechanisms of ZnO NPs to kill bacteria and eradicate biofilms, as there is a single particular phenomenon that governs the activity of ZnO NPs. Our study demonstrates a promising approach to develop effective solutions for combatting in vitro drug-resistant bacterial infections. We need to assess this in relevant animal models to validate the effectiveness of ZnO NPs in potentiating gentamicin activity.

## Data Availability

The original contributions presented in the study are included in the article/Supplementary Material; further inquiries can be directed to the corresponding author.

## Supplementary Materials

The following supporting information can be downloaded at: https://www.mdpi.com/article/10.3390/pharmaceutics18080913/s1, Table S1: Minimum Inhibitory Concentration and Minimum Bactericidal Concentration of Gentamicin against different bacterial strains, Table S2: Diplazium esculentum plant extract characterization (top 10 most abundant metabolites), Figure S1: Antibacterial activity of ZnO nanoparticles against clinical isolates of *Klebsiella pneumoniae*.
