# Supplementary material for: Potentiating Gentamicin Efficacy Against Biofilms of Clinically Relevant Gram-Negative Bacteria Using Biosynthesized ZnO Nanoparticles
Source: Pharmaceutics. 2026 Jul 24;18(8):913. doi: 10.3390/pharmaceutics18080913 (PMC13516594; doi:10.3390/pharmaceutics18080913)
Supplement: Supplementary file 1 [file pharmaceutics-18-00913-s001.zip › pharmaceutics-4440034-supplementary.pdf]

Supplementary Information

**Bacterial Strain**	**MIC Concentration (µg/ml)**	**MBC Concentration (µg/ml)**	**Sub-MIC Concentration (µg/ml)**
**ECU-6**	**3.125**	3.125	**1.5625**
**SAW-1**	**6.25**	`12.5	**3.125**
**KPP-1**	**1.5625**	1.5625	**0.78125**

Table S1: Minimum Inhibitory Concentration and Minimum Bactericidal Concentration of Gentamicin against different bacterial strains

Metabolite Name	Formula	Class/Significance	Abundance
Cucurbitacin E	$C_{32}H_{44}O_8$	Triterpenoid (Bioactive)	236,758,192
Eudesmin	$C_{22}H_{26}O_6$	Lignan (Antioxidant)	35,762,076
(+)-Sabinene	$C_{10}H_{16}$	Monoterpene	2,542,591
4-Hydroxycoumarin	$C_9H_6O_3$	Coumarin derivative	1,781,774
Glucohesperalin	$C_{14}H_{27}NO_{10}S_3$	Glucosinolate derivative	1,360,900
Cholesteryl β-D-glucoside	$C_{33}H_{56}O_6$	Sterol glycoside	1,170,080
4-Prenylresveratrol	$C_{19}H_{20}O_3$	Prenylated Stilbene	944,210
Eriodictyol 7-(6-galloylglucoside)	$C_{28}H_{26}O_{15}$	Flavonoid Glycoside	736,524
6-Gingerol	$C_{17}H_{26}O_4$	Phenolic (Ginger derivative)	689,865
Shikimic acid	$C_7H_{10}O_5$	Plant pathway precursor	550,211

Table S2: *Diplazium esculentum* plant extract characterization (Top 10 most abundant metabolites).

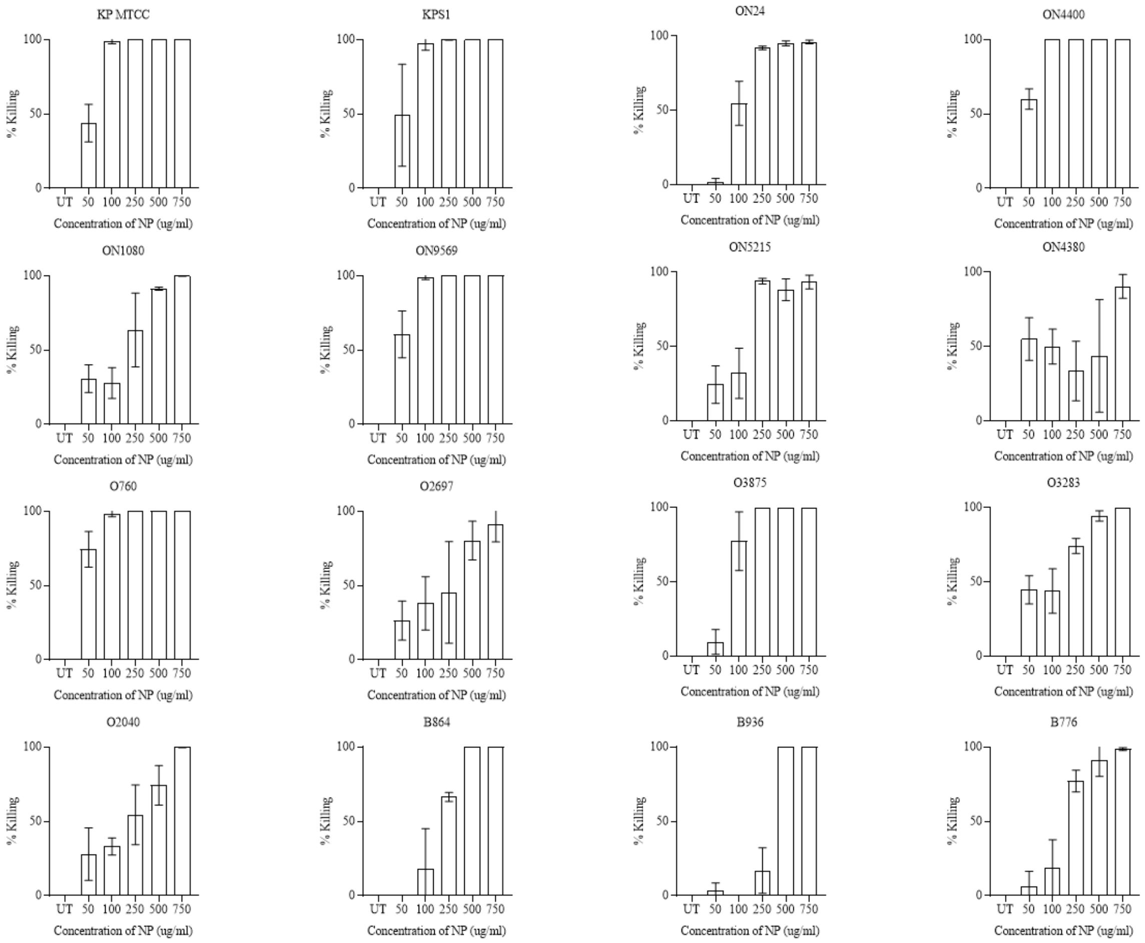

Figure S1: Antibacterial activity of ZnO nanoparticles against clinical isolates of *Klebsiella pneumoniae*. The percentage of viable cell reduction of *K. pneumoniae* MTCC 10309 and multiple clinical isolates (KPS1, ON24, ON4400, ON1080, ON9569, ON5215, ON4380, O760, O2697, O3875, O3283, O2040, B864, B936, and B776) following 24 h exposure to increasing concentrations of ZnO nanoparticles (50–750 µg/mL). Untreated cultures (UT) served as control. Percentage killing was calculated based on the reduction in CFU/mL relative to untreated samples. Data are presented as mean ± SD from three independent experiments (n = 3). A concentration-dependent antibacterial response was observed across most isolates, with near-complete killing at higher ZnO NP concentrations, highlighting isolate-specific susceptibility profiles.
